# Geographical Variations in Sex Ratio Trends over Time in Multiple Sclerosis

**DOI:** 10.1371/journal.pone.0048078

**Published:** 2012-10-25

**Authors:** Maria Trojano, Guglielmo Lucchese, Giusi Graziano, Bruce V. Taylor, Steve Simpson, Vito Lepore, Francois Grand’Maison, Pierre Duquette, Guillermo Izquierdo, Pierre Grammond, Maria Pia Amato, Roberto Bergamaschi, Giorgio Giuliani, Cavit Boz, Raymond Hupperts, Vincent Van Pesch, Jeannette Lechner-Scott, Edgardo Cristiano, Marcela Fiol, Celia Oreja-Guevara, Maria Laura Saladino, Freek Verheul, Mark Slee, Damiano Paolicelli, Carla Tortorella, Mariangela D’Onghia, Pietro Iaffaldano, Vita Direnzo, Helmut Butzkueven, MSBase Study Group and the New Zealand MS Prevalence Study Group

**Affiliations:** 1 Department of Neuroscience and Sense Organs, University of Bari, Bari, Italy; 2 Department of Clinical Pharmacology and Epidemiology, Consorzio Mario Negri Sud, S. Maria Imbaro, Italy; 3 Menzies Research Institute, University of Tasmania, Hobart, Australia; 4 Neuro Rive-Sud, Hôpital Charles LeMoyne, Quebec, Canada; 5 Hôpital Notre Dame, Montreal, Canada; 6 Hospital Universitario, Sevilla, Spain; 7 Hotel-Dieu de Levis, Quebec, Canada; 8 Department of Neurology University of Florence, Florence, Italy; 9 Neurological Institute IRCCS Mondino, Pavia, Italy; 10 Ospedale di Macerata, Macerata, Italy; 11 Karadeniz Technical University, Trabzon, Turkey; 12 Maaslandziekenhuis, Sittard, The Netherlands; 13 Cliniques Universitaires Saint-Luc, Brussels, Belgium; 14 John Hunter Hospital, New Lambton Heights, New South Wales, Australia; 15 Hospital Italiano, Buenos Aires, Argentina; 16 FLENI, Buenos Aires, Argentina; 17 University Hospital La Paz, IdiPAZ, Madrid Spain; 18 INEBA, Buenos Aires, Argentina; 19 Groen Hart Ziekenhuis, Gouda, Netherlands; 20 Flinders University and Medical Centre, Adelaide, South Australia, Australia; 21 Department of Neurology, Royal Melbourne Hospital, Parkville, Victoria, Australia; 22 Department of Medicine, The University of Melbourne, Melbourne, Victoria, Australia; 23 Department of Neurology, Box Hill Hospital, Monash University, Melbourne, Victoria, Australia; Charité University Medicine Berlin, Germany

## Abstract

**Background:**

A female/male (F/M) ratio increase over time in multiple sclerosis (MS) patients was demonstrated in many countries around the world. So far, a direct comparison of sex ratio time-trends among MS populations from different geographical areas was not carried out.

**Objective:**

In this paper we assessed and compared sex ratio trends, over a 60-year span, in MS populations belonging to different latitudinal areas.

**Methods:**

Data of a cohort of 15,996 (F = 11,290; M = 4,706) definite MS with birth years ranging from 1930 to 1989 were extracted from the international MSBase registry and the New Zealand MS database. Gender ratios were calculated by six decades based on year of birth and were adjusted for the F/M born-alive ratio derived from the respective national registries of births.

**Results:**

Adjusted sex ratios showed a significant increase from the first to the last decade in the whole MS sample (from 2.35 to 2.73; p = 0.03) and in the subgroups belonging to the areas between 83° N and 45° N (from 1.93 to 4.55; p<0.0001) and between 45° N to 35° N (from 1.46 to 2.30; p<0.05) latitude, while a sex ratio stability over time was found in the subgroup from areas between 12° S and 55° S latitude. The sex ratio increase mainly affected relapsing-remitting (RR) MS.

**Conclusions:**

Our results confirm a general sex ratio increase over time in RRMS and also demonstrate a latitudinal gradient of this increase. These findings add useful information for planning case-control studies aimed to explore sex-related factors responsible for MS development.

## Introduction

Multiple Sclerosis (MS) is a chronic demyelinating immune-mediated disease of the Central Nervous System. The aetiology of MS is subject to an extensive research effort and remains undetermined. The importance of genetic factors in susceptibility to MS is supported by several genetic epidemiological studies [1], whereas migration studies, geographical gradients and disease discordance in identical twins indicate that environmental factors [2]–[3] could influence the development of MS, and that gene-environment interactions may exert an even stronger effect. [4]–[5].

Recent meta-analyses [6] of epidemiological data have highlighted some relevant trends over the past few decades. A general increase in the prevalence and incidence of MS, over time, was demonstrated in many countries around the world, suggesting that, whatever the causative factors of MS may be, their influence appears to increase. A flattening of the latitudinal prevalence gradient in MS in Europe and North America was found, suggesting a gradual alteration of latitude-associated risk of MS. [6]–[7] However results derived from data collected at different times in distant regions and countries, are of major methodological concern in regards to the variability in population size, composition, ethnic origin and age. Moreover, increasing frequency of MS could be ascribed, at least partially, in some countries, to a global improvement of case ascertainment, [7]–[8] diagnosis being dependent on accessibility to adequate medical structures and personnel, and to the availability of more sensitive paraclinical tools which have been included in the new MS diagnostic criteria. [9]–[10] As a consequence of this variability, it is very difficult to aggregate and compare data from different studies. Therefore it is becoming evident that new epidemiological approaches are needed to evaluate the changes in incidence and the geographical distribution of the disease.

The recently proposed “sex ratio methodology” [11] has opened new perspectives on MS epidemiology. Indeed the female/male (F/M) sex ratio is a robust epidemiological marker and can be considered as an internal variable in the same study groups, thus improving aggregation and comparison of data collected in different countries and regions. Moreover, confounding effects exerted by differences in time to diagnosis and age of onset between sexes can be minimized by grouping patients by the year-of-birth approach. [11]–[12].

Recent studies designed to investigate change in F/M sex ratio in MS populations showed an increase over time in several countries, [11], [13]–[15] regions [16] and cities. [17]–[18] This increase mainly affected patients with relapsing onset MS (RRMS). [17], [19] More recent data from other high MS prevalence areas, such as New Zealand [20] and Tasmania, [21] have shown sex ratio stability over time.

To date, no study has been designed and conducted in order to directly compare the sex ratio trends over time among MS populations from different geographical areas. Difficulties in uniformly collecting data and handling large different datasets [22] undoubtedly constitute a major obstacle in such a direction. The establishment, since July 2004, of the international MSBase internet platform, [23] with a shared management system, allowed to overcome at least some of the limitations of regional variability. The MSBase platform provides an excellent opportunity for international, collaborative epidemiological studies of MS. On the basis of the availability of a large dataset, uniformly collected in this database, through a standardized software system (iMed), we carried out an exploratory analysis aimed to assess and compare the significance of year of birth as predictor of sex ratio in MS populations from different worldwide geographical areas, over a 60-year span. New Zealand data, deriving from a nationwide prevalence survey conducted in 2006, [20] were also included in this analysis.

## Methods

### Cohort Definition and Methods

The MSBase Registry is a strictly observational clinic based database established in July 2004 for sharing, tracking and evaluating outcomes data in MS. Investigators aim to include either all patients or all newly diagnosed patients into the database. Data are collected in each participating center by a standardized database management system (iMed). Anonymised datasets are periodically uploaded to the MSBase server. Objectives, methods and details of the MSBase project have previously been described. [23] Data for the current analysis were extracted from the international MSBase registry in April 2009 and additional data were provided by the New Zealand MS database. [20] The New Zealand data comes from a national prevalence survey, with data validation from treating neurologists. Although both datasets are collected with different methodologies, accurate recording of year of symptom onset and sex occur in both, so that combination of the datasets is reasonable for the current analysis.

A cohort of 15,996 (F = 11,290; M = 4,706) patients with a definite MS according to Poser, [24] McDonald or Polman criteria [9]–[10] and with birth years ranging from 1930 to 1989, was selected. Patients born before 1930 and after 1989 were not included in the study because the number of patients was comparatively small. The mean numbers of women and men by year of birth (yob) were averaged over 10-year blocks to calculate the sex ratio. Each block included a mean of 2,658 cases.

Mean age at onset, mean time from onset to diagnosis and frequency of mono-symptomatic or poly-symptomatic onset were evaluated according to sex.

Sex ratio trends were analyzed in the whole MS sample and, separately, after a stratification by geographical areas. A comparison among data from three large groups of countries belonging to different latitudes (northern area from 83° N and 45° N , intermediate area from 45° N to 35° N and southern area from 12° S and 55° S) and, in the smaller European subgroup, between Northern and Southern countries was performed. A complete list of countries which provided at least 100 cases is shown in Table 1.

**Table 1:** List of Countries with over one hundred cases, divided into three latitude areas.

NORTHERN (83° N - 45° N)		CENTRAL (<45^o^N-35^o^N)		SOUTHERN (12° S - 55° S)	
*Country*	*n patients*	*Country*	*n patients*	*Country*	*n patients*
Belgium	276	France	171	Argentina	706
Canada	1995	Italy	4293	Australia	1664
Germany	151	Portugal	290	New Zealand	2810
Denmark	397	Spain	711		
The Netherlands	1316	Turkey	354		
TOTAL	4135	TOTAL	5819	TOTAL	5180

Analyses of gender ratio trends were carried out also after a stratification of patients by RR or primary progressive (PP) initial clinical course.

### Patient Consent and Ethics

Ethics Committee approval and written signed informed consent from patients were required and obtained for participation at each contributing centre.

### Statistical Analyses

The mean age at onset and the mean time from onset to diagnosis in different groups of patients were compared using the Mann-Withney *U* test.

The F/M ratios were calculated using a multivariate logistic regression, per six decades by the yob approach. To avoid a potential bias from gender disequilibrium at birth, ratios were adjusted for the F/M born-alive ratio (bar) derived from National Registries of births in the respective native Countries. In particular, the true proportion (P_y_) of women with MS born in each year (y) was modeled as: ln[P_y_/(1– P_y_)] = βintercept+β1[yob]+β2[bar]. With each year of birth the F/M ratio increased by a factor of e^β1[yob]^. All the analyses were performed using the Statistical Analysis System (SAS) Package, Release 9.1.

## Results

Table 2 shows the distribution of patients by 10-year intervals based on year of birth. F/M ratio showed a progressive increase from the first to the last decade (from 2.35 to 2.73; p-value for trend = 0.032) by a factor (eβ1[yob]) of 1.003 with each year of birth. The frequency of RR course was significantly ( p<0·05) higher and the mean age at onset was significantly (p<0·05) earlier in female than in male patients in all the decades examined. The mean time from onset to diagnosis declined during the six decades under evaluation (p<0·001) in both female (from 8.96 to 1.69 years) and male ( from 8.74 to 1.78 years) patients, but it did not differ between sexes in the whole cohort and in the single decades examined (Table 2). Similar trends of F/M ratio, age at onset, RR course at onset, time from onset to diagnosis were found in the three subgroups of patients belonging to northern, intermediate and southern latitudes (Table 3).

**Table 2:** Sex ratio, percent of relapsing onset, mean age at onset and mean time from onset to diagnosis by birth decades in female (F) and male (M) MS patients.

Decadesof birth	Ratio	Type course% RR/RR+PP^Λ^by sex		Mean Age at onset by sex°		Mean Time from onsetto diagnosis by sex°	
	F/M*	F	M	F	M	F	M
1930–1939	460/196	73.21	52.02§	42.88±13.01	44.64±11.83	8.96±10.62	8.74±9.97
	2.35						
1940–1949	1461/641	79.26	66.84§	39.88±11.28	41.27±10.80 §	7.32±8.77	6.48±7.54
	2.28						
1950–1959	2707/1161	89.52	79.50§	35.87±9.17	36.71±8.97 §	5.57±6.60	5.57±9.11
	2.33						
1960–1969	3340/1391	94.86	89.78§	30.95±7.18	31.54±7.20 §	3.94±4.58	3.61±4.42
	2.40						
1970–1979	2494/1014	97.98	95.18	25.41±5.17	25.96±4.96 §	2.60±3.31	2.42±3.28
	2.46						
1980–1989	828/303	98.95	97.87	19.82±3.68	19.60±4.21	1.69±2.08	1.78±2.23
	2.73						
Total	11290/470606	91.66	84.23§	32.03±10.24	32.97±10.27 §	4.61±6.24	4.39±6.70
	2.41						

* *15996 pts; p-value for trend 0.032; ^Λ^14543 pts; °11998 pts; § p<0.05 sex M vs. F.*

RR = Relapsing remitting; PP = Primary progressive.

**Table 3:** Sex ratio, percent of relapsing onset, mean age at onset and mean time from onset to diagnosis by birth decades and latitude in female (F) and male (M) MS patients.

Northern Latitude		Type course%RR/RR+PP^Λ^		Mean Age at onset by sex°		Mean Time from onset to diagnosis by sex°	
Decade of birth	F/M*	F	M	F	M	F	M
1930–1939	87/45	70.27	62.79	43.33±13.40	41.38±13.83	8.61±9.77	9.43±11.38
	1.93						
1940–1949	370/171	75.44	62.73	40.08±11.15	41.94±10.59	7.46±8.36	5.64±6.65§
	2.16						
1950–1959	792/327	88.69	79.08	35.69±9.31	36.27±8.91	5.39±6.38	5.26±5.78
	2.42						
1960–1969	932/369	95.65	88.89	30.92±7.32	31.77±6.94	3.80±4.42	3.46±4.19
	2.53						
1970–1979	625/173	97.82	91.19	25.80±5.07	26.77±4.59 §	2.58±3.09	2.15±2.68
	3.61						
1980–1989	200/44	98.93	100.00	20.24±3.35	20.38±4.68	1.75±2.12	2.32±2.93
	4.55						
TOTAL	3006/1129	91.36	81.84	31.92±9.91	33.67±9.77 §	4.42±5.79	4.25±5.50
	2.66						
*4135 pts; p-value for trend 0.0001; ?3877 pts; °3619 pts; § p<0.05 sex M vs. F							

LatitudeNorth-Central		Type course%RR/RR+PP^Λ^		Mean Age at onset by sex°		Mean Time from onset to North-Central	
Decade of birth	F/M*	F	M	F	M	F	M
1930–1939	89/61	67.47	54.24	46.33±12.58	43.20±11.12	8.41±9.63	9.03±10.50
	1.46						
1940–1949	340/174	76.45	72.02	39.78±11.75	40.91±12.11	8.30±9.59	8.46±9.68
	1.95						
1950–1959	734/382	90.68	82.66	35.26±9.05	36.47±9.54	6.94±7.22	6.23±7.23
	1.92						
1960–1969	1247/579	95.02	91.64	30.52±7.23	30.26±7.67	4.20±4.81	4.23±5.12
	2.15						
1970–1979	1082/534	98.02	97.12	24.94±5.49	25.26±5.33	2.92±3.82	2.60±3.73
	2.03						
1980–1989	416/181	98.54	98.86	19.35±3.77	19.20±4.20	1.78±2.05	1.75±2.28
	2.30						
TOTAL	3908/1911	93.23	89.11	29.66±9.82	30.18±10.15	4.51±5.96	4.40±6.16
	2.05						
*5649 pts; p-value for trend 0.0425; ?5649 pts; °3487pts							

Latitude Southern		Type course%RR/RR+PP^Λ^		Mean Age at onset by sex°		Mean Time from onset to diagnosis by sex°	
Decade of birth	F/M*	F	M	F	M	F	M
1930–1939	270/77	75.86	43.66	42.08±12.90	46.81±10.83 §	9.17±11.08	8.30±9.10
	3.51						
1940–1949	690/268	82.54	66.03	39.83±11.21	41.03±10.45	6.95±8.67	6.26±7.04
	2.57						
1950–1959	1027/388	89.32	76.74	36.26±9.11	37.17±8.66	5.10±6.39	5.41±11.69
	2.65						
1960–1969	973/355	93.94	87.61	31.31±6.98	32.58±6.80 §	3.86±4.54	3.15±3.79§
	2.74						
1970–1979	651/246	98.08	93.56	25.60±4.83	26.45±4.48 §	2.27±2.82	2.34±2.90
	2.65						
1980–1989	168/67	100.00	93.65	20.26±3.79	19.86±3.86	1.41±2.06	1.47±1.41
	2.51						
TOTAL	3779/1401	90.26	79.36	33.69±10.44	34.81±10.24 §	4.81±6.72	4.49±7.84
	2.70						
*5180 pts; ?5017 pts; °4892 pts; § p<0.05 sex M vs. F							

RR = Relapsing remitting; PP = Primary progressive.

We also assessed the frequencies of mono-symptomatic and poly-symptomatic onset in female and male patients. The results showed that they did not differ (p = 0.36) between genders either in the whole sample (82.2% and 10.7% in females; 88.7% and 11.2% in males) or in the single decade cohorts examined. Moreover the time from onset to diagnosis was similar in females and males belonging to the mono-symptomatic (3.08 ± 5.48; 3.15 ± 5.58 years, respectively) or poly-symptomatic (4.03 ± 6.97; 3.97 ± 12.32 years, respectively) groups.

**Figure 1:**
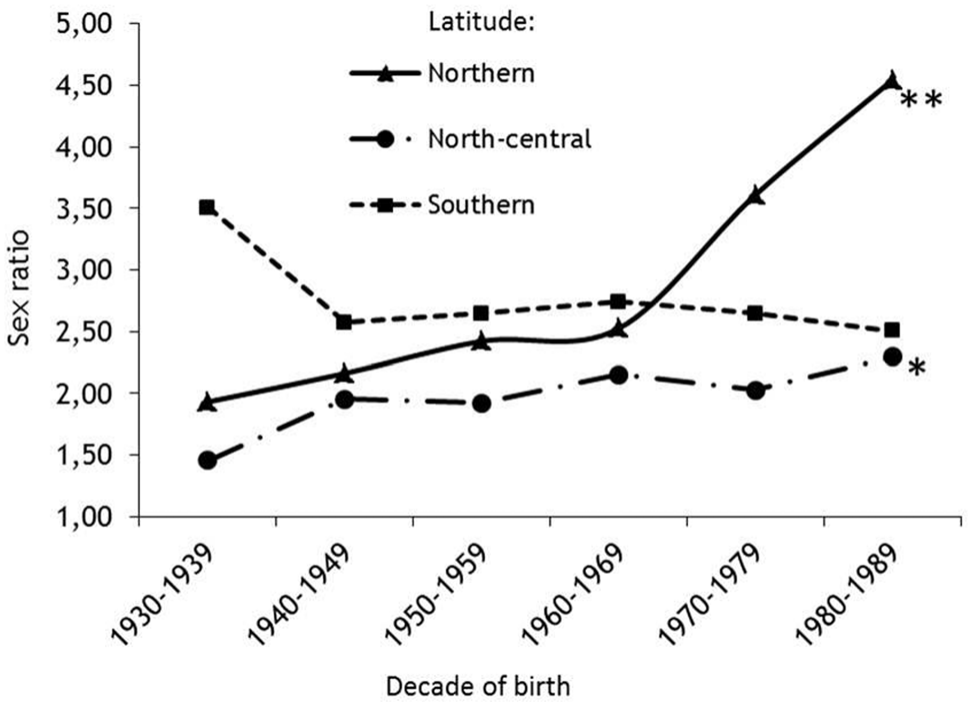
Plot of gender ratio by six birth decades in MS patients stratified by Latitude. p-value for trend *0.0425; **<0.0001.

**Figure 2:**
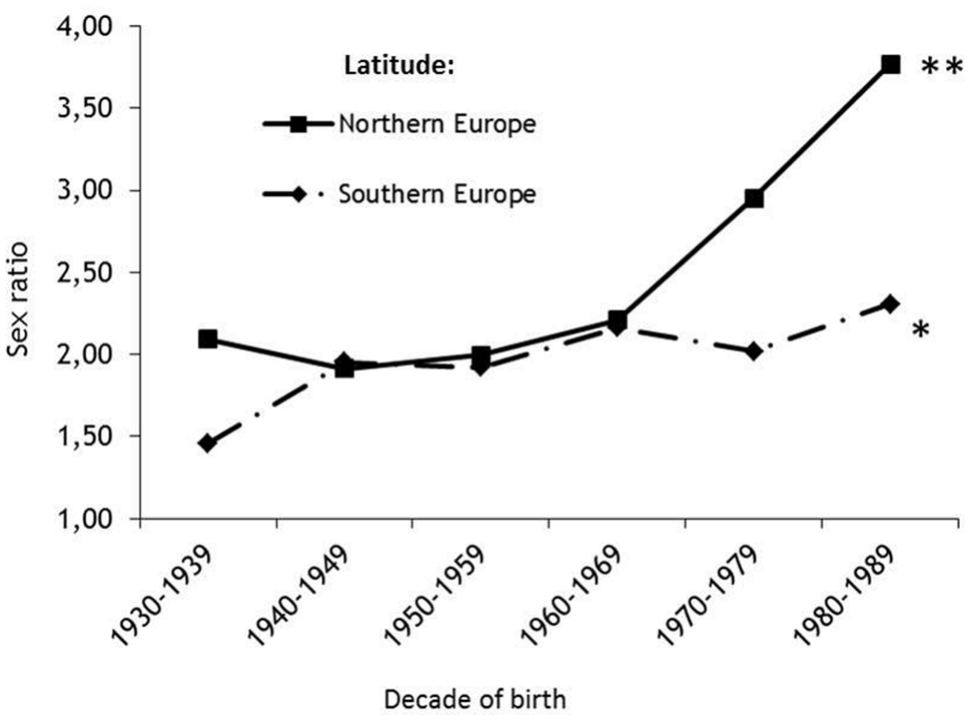
Plot of gender ratio by six birth decades in MS patients from Northern and Southern Europe. p-value for trend *0.0426; **<0.0004.

In the analysis by latitude (Fig. 1), the adjusted F/M ratio showed a progressive increase, through sixty years, from 1.93 to 4.55 (p<0.0001) by a factor (eβ1[yob]) of 1.016 with each year of birth and with an apparent peak of increase after the 1970s, in the area from 83° N and 45° N. The sex ratio also increased from 1.46 to 2.30 (p<0.05) with a factor (eβ1[yob]) of 1.005 with each year of birth in the area between 45° N and 35° N, whereas no significant relationship was found between sex ratio and year of birth at latitudes between 12° S and 55° S where the F/M values remained steadily high over time (from 3.51 to 2.51; p = 0.355),

The analysis was performed, also, in the subgroup of European patients (N.7959), separately (Fig.2). A substantial increase of the F/M ratio from 2.09 to 3.77 (p<0.0004), with an increasing factor (eβ1[yob]) of 1.014 for each year of birth, was found in Northern Europe (N.2140 patients), whereas only a moderate increase from 1.46 to 2.31 (p<0.05) with an annual factor (eβ1[yob]) of 1.005 was shown in Southern Europe (N.5819 patients).

**Figure 3:**
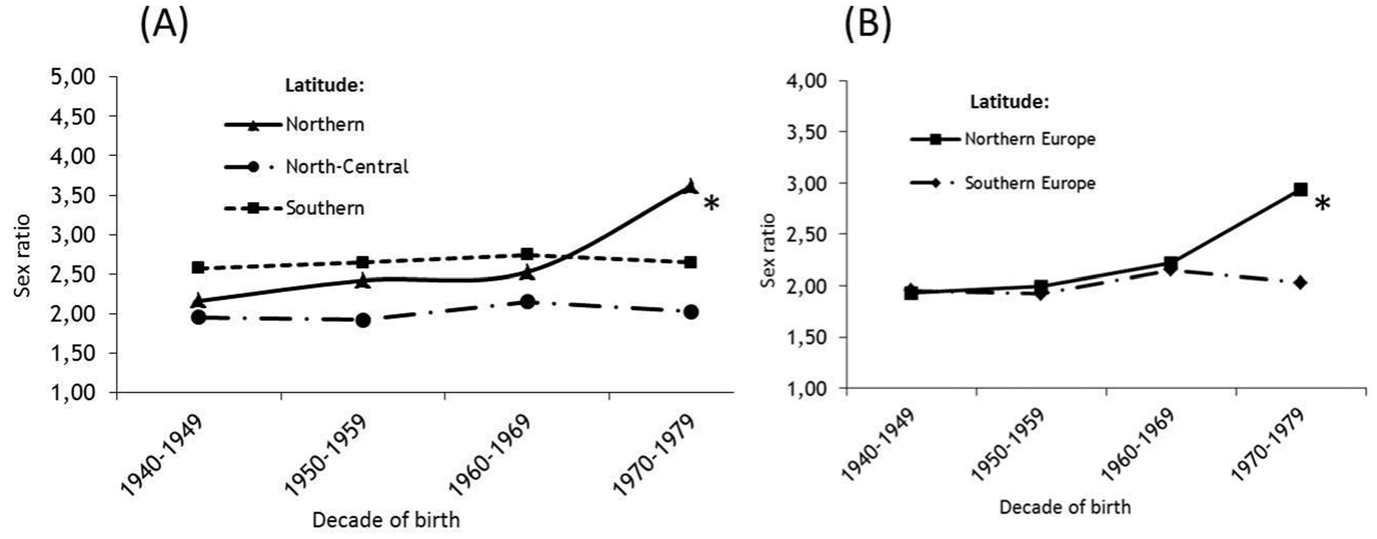
Plot of gender ratio by four birth decades in MS patients stratified by Latitude (A) and from Northern and Southern Europe (B). p-value for trend *<0.001.

In order to verify the consistency of these results we repeated the sex ratio analysis by latitude after the exclusion of patients belonging to the first (1930–1939) and the last (1980–1989) birth cohorts who could cause a spurious trend in sex ratio mainly because of younger survival age^25^ and age at onset in females than in males. The results paralleled the results of the entire cohort (Fig 3 A- 3B) confirming a more significant increase in the areas belonging to northern latitudes both worldwilde (p<0.001) and in Europe (p = 0.0051).

**Figure 4:**
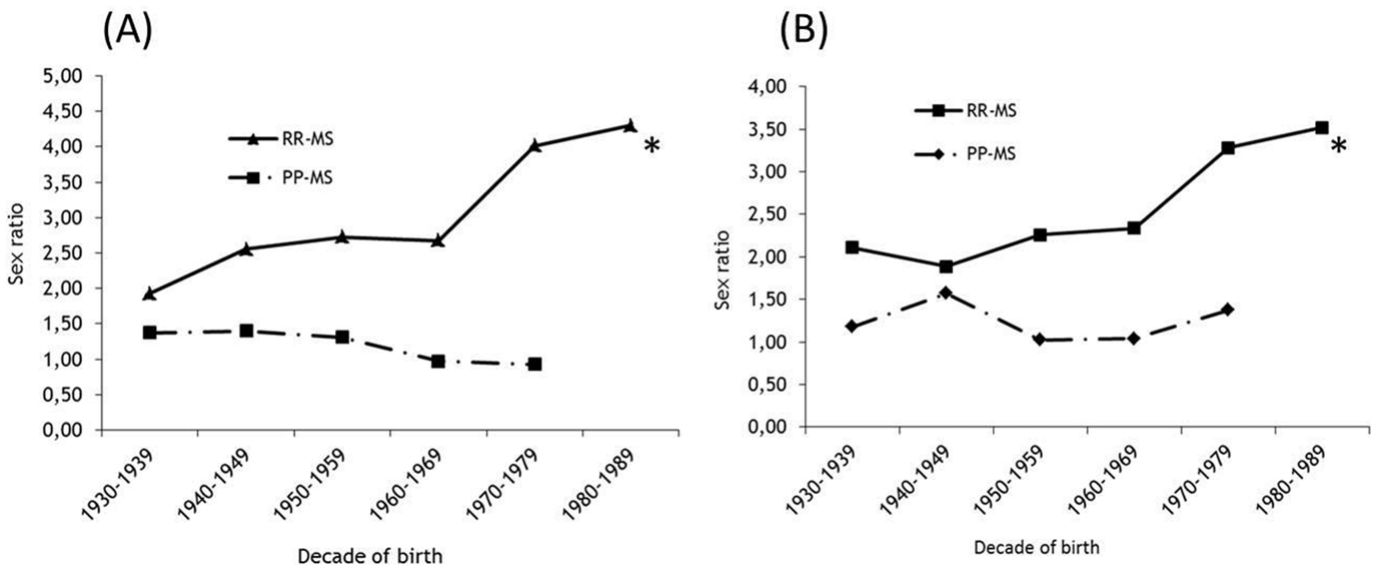
Plot of gender ratio by six birth decades in MS patients from Northern Latitude Area (A) and Northern Europe (B) stratified by Relapsing Remitting (RR) and Primary Progressive (PP) disease course. p-value for trend *<0.001.

Finally the sex ratio analysis by latitude was carried out after a stratification of patients by disease course (RR or PP) at onset in the countries belonging to northern latitude areas and to Northern Europe, the results showed that the remarkable increase in ratio was distributed among patients with a RR course (Fig. 4 A and 4 B). The values ranged from 1.93 to 4.30 (p<0.0001) with an increasing factor (eβ1[yob]) of 1.014 and from 2.11 to 3.52 (p = 0.0016) with an increasing factor (eβ1[yob]) of 1.015 in the global Northern and Northern European samples, respectively. No change in ratio was observed for patients with PP course in both samples.

## Discussion

We conducted the first direct comparison of the sex ratio trends, evaluated by a yob approach [11]–[12] over a 60-year span, among MS populations from different latitudes. The analysis was facilitated by the availability of a large international database with shared demographic and clinical information collection. Several tests were conducted to identify potential confounders that could materially influence the results. Firstly, to control for a bias due to a possible sex disequilibrium at birth, the F/M ratios of MS patients were adjusted for the F/M born-alive ratios derived from national birth registries in the respective native participating countries. Secondly, to exclude that a differential access to specialized medical care structures for females and males, over time, in different countries, could be responsible of an apparent increase of F/M ratio, we calculated the time elapsed from onset to diagnosis during the six decades under evaluation in the two sexes. In both female and male groups, time to diagnosis declined over time, probably explained by the increased awareness of the disease and new diagnostic tools, [9]–[10] however this time did not differ by sex in the different decades examined, indicating that any changes over time in ascertainment, accessibility to medical structures and procedures were equally applicable to both sexes and without significant differences among countries. Similar trends of F/M ratio, age at onset, RR course at onset, time from onset to diagnosis were found in the three subgroups of patients belonging to northern, intermediate and southern latitudes Thirdly, another potential bias [26] is based on the assumption that milder onset forms of the disease, potentially more common in females, are currently more readily diagnosed as MS than in the past, we investigated whether the frequency of the relatively milder mono-symptomatic and the more severe poly-symptomatic onset differed by sex, but no differences were found, suggesting a lack of diagnostic ascertainment bias on the basis of disease onset severity.

Our results confirm a general, though modest, increase of the F/M sex ratio (from 2.35 to 2.73) over time among patients affected by MS, but also show that the rate of increase varies with latitude. The highest values were observed in countries located in the most polar latitudes, whereas only a slight increase was observed in countries of the intermediate latitude, and no increase in sex ratio was found in countries located at the most southern latitudes. Similar trends were observed when the analysis was limited to the smaller European sample.

The consistency of these results was also confirmed when the analysis was restricted to the four central decades of birth after the exclusion of the oldest and youngest patients belonging to the first and last decades who could bias the trend in sex ratio, mainly because of younger survival age [25] and age at onset in females than in males and of an heavy-right truncation of the follow-up in the later birth cohort.

Our findings are consistent with the results from previous studies conducted in single countries, investigating sex ratio changes in time spans comparable to those we examined. The trend of increase (from 1.9 in 1931 to 3.21 in 1980) found in Canada, [11] in the largest population studied so far, and in Oslo, Norway, [17] (from 1.48 in 1910 to 2.30 in 1979) is comparable to our results from all Northern latitude countries (from 1.96 in 1930 to 4.55 in 1989) and from Northern European countries (2.09 in 1930 to 3.77 in 1989). On the other hand, recent studies from New Zealand [20] and Tasmania [21] found that high F/M sex ratios remained stable over time (since birth year 1940) as we found on a larger scale in most equatorial latitude countries where the F/M ratio remained steadily equal to or higher than 2.5 in all the analysed decades.

Our results also demonstrate that the global increase of MS incidence in polar and intermediate latitudes is principally driven, as previously demonstrated in other studies, by females with RRMS. [17]–[19] This increase in MS incidence may result from an intensification of the effect, in these latitudes, of sex-related factors, the influence of which seems otherwise unchanged over time in latitudes where high sex ratio disequilibrium remains stable. The kind of etiological factor(s) causing increased disease susceptibility in women can only be speculated upon. [27] Given the short duration over which sex ratio changes have occurred, genetic factors are unlikely to be driving this change. [11] It is more likely that the observed change in sex ratio is due to altered environmental factors or the result of latitudinally-sensitive gene-environment interactions. This hypothesis has also been supported by the results of a recent study [28]showing that the sex ratio in immigrants to Canada, with post-migration MS onset, is increasing. The rate of the increase observed in this study was the highest in immigrants of southern-European origin by a factor of 1.27 per 10-year period. This rate of increase was higher than that which we found in the MS population born and resident in southern European countries (annual factor of 1.005), suggesting that moving to Canada increases MS risk in women of southern European origin.

However, latitudinal effects cannot uniquely explain regional variation in female MS incidence. Interestingly, a remarkable increase in the sex ratio was found in Isfahan, [18] an Iranian province approximately located on the border between the northern and intermediate latitude areas, as defined in our study, where minimal, if any, increase in sex ratio could be expected on the basis of our results. These results suggest that latitudinal variations of disease frequency may be modified by the effect of other concurrent risk factors (ethnic, cultural and socio-economic).

There is growing evidence that solar ultraviolet radiation (UV) exposure, which varies with latitude, is associated with vitamin D biological activity and sex in contributing to the risk of MS [29]–[31] and higher sunlight exposure before the age of 15 has been associated with a decreased risk of MS. [32] In a study carried out in the province of Isfahan, [33] the prevalence of severe vitamin D deficiency (<8 ng/ml) in winter was significantly higher in high school-age girls than in boys. The 1,25-dihydroxyvitamin D3 or calcitriol has a well recognized modulating effect on the adaptive immune system and particularly on T cell homeostasis. [34] Immunomodulation exerted by calcitriol seems to be biased towards female sex in its protective effect both in the experimental animal model of MS [35] and in MS patients. [36]–[37] A dose-dependent and linear correlation between increasing levels of vitamin D and reduced hazard of relapse in people with RR-MS was recently reported [38] and a synergism between 17β estradiol and vitamin D3, leading to an increased anti-inflammatory and protective effect of vitamin D3 in women, [37] has also been demonstrated.

Even more intriguing is the fact that solar exposure is not only related to latitude but also to cultural and social aspects. Studies carried out in countries with Islamic tradition have shown that vitamin D deficiency is more common in veiled women. [39] Vitamin D serum levels also depend on alimentary intake, again an extremely variable element either among different countries as among single individuals. Furthermore changes in other environmental risk factors for MS such as smoking behaviour [40]–[41] and infections [42] could interact with physiological differences between genders, such as sex steroids or sex-related gene-expression and epigenetic regulation, converging together with Vitamin D activity, on molecular immunological and inflammatory mechanisms that have been shown to have a sex bias. [43]–[45] This quite complex congregation of variables could well account for exceptions that can be found to the latitudinal gradient principle.

It seems likely, ultimately, that latitudinal, genetic and local environmental factors interact to cause MS, and our study confirms that the latitudinal effects are becoming more relevant at polar and intermediate latitudes for the RR phenotype in women.

In conclusion this exploratory analysis on the geographical variations in sex ratio trends over time in MS does not claim to have the strength of a global population-based study, nevertheless some significant remarks can be drawn from the results. First, our findings encourage further use of the MS sex ratio methodology, to reappraise its trend in the next years on a prospective basis and to validate its relationship with more traditional epidemiological measures such as incidence and prevalence. Second, the demonstration of an uneven distribution of sex ratio time-trends among populations from different geographical areas, derived by a direct comparison of uniformly collected data in an international database, adds useful information for planning studies aimed to explore the paths of aetiological hypotheses about MS, primarily the role of Vitamin D. This should be effectively done both by case-control studies as well as by focused bench and in vivo research. The mergence of both these epidemiological “top-down” and laboratory “bottom-up” approaches could return huge clinical advantages enhancing prevention and treatment of MS.
